# Targeted Lipidomics Reveals *Citrus aurantifolia* Peel Extract's Potential to Increase Lipid Catabolism With PC34:1 as a Discriminative Metabolite

**DOI:** 10.1002/fsn3.72108

**Published:** 2026-07-20

**Authors:** Pakkapong Phucharoenrak, Kemika Praengam, Dunyaporn Trachootham

**Affiliations:** ^1^ Institute of Nutrition Mahidol University Nakhon Pathom Thailand

**Keywords:** acylcarnitine, functional food, lime peel extract, lipid metabolism, liver, metabolomics

## Abstract

Regulating lipid metabolism is the main mechanism of exercise to control blood lipids, body weight, and prevent metabolic diseases. However, the effects of complex mixtures of phytochemicals, particularly from plant‐derived extracts, on lipid metabolism remain poorly understood. Recently, we developed a green extraction method for lime ( *Citrus aurantifolia* ) peel, yielding an extract rich in three major compounds (hesperidin, limonin, and bergaptol). In this study, the effect of the extract on lipid metabolism was investigated in normal human hepatocytes (THLE‐2) using a targeted lipidomics approach under nontoxic conditions. The results showed significant increases in 75 lipid metabolites and decreases in four metabolites (propenoylcarnitine [C3:1], octadecadienylcarnitine [C18:2], PC 42:1, and dodecenoylcarnitine). Interestingly, metabolites showing ≥ 2‐fold changes were exclusively acylcarnitines, with increases in medium‐chain acylcarnitines (C12 and C6:1) and a decrease in long‐chain acylcarnitine (C18:2), consistent with altered fatty acid metabolism. A significant fourfold decrease in C3:1 indicates reduced conversion of branch‐chain amino acids (isoleucine) to fatty acid intermediates. PCA, PLS‐DA, and OPLS‐DA analyses consistently identified phosphatidylcholine 34:1 (PC34:1) as the key metabolite distinguishing the treated and control groups, based on VIP score. Taken together, the findings suggest that lime peel extract may reduce lipid synthesis from amino acids and may promote lipid catabolism in hepatocyte cell models with a dominant medium‐chain acylcarnitine pattern, and PC34:1 as the discriminative metabolite associated with treatment response. This novel functional ingredient may have potential implications in preventing dyslipidemia and metabolic dysfunction‐associated steatotic liver disease (MASLD), warranting further in vivo and clinical studies.

## Graphical abstract

Targeted lipidomics reveals a dominant increase in medium‐chain acylcarnitines after normal liver cells are exposed to the limonin‐hesperidin‐rich *Citrus aurantifolia* peel extract. This pattern of increased β‐oxidation is similar to that observed during exercise. Also, the lime peel extract reduced the conversion of branched‐chain amino acids to fatty acid intermediates. PCA, PLS‐DA, and OPLS‐DA consistently revealed PC 34:1 as the most prominent discriminative metabolite. These findings suggest the potential of lime peel extract to increase lipid catabolism and prevent MASLD.

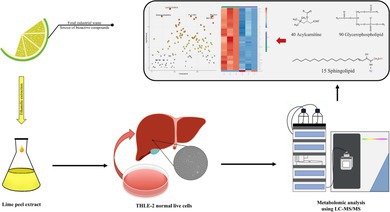

## 1 Introduction

Lipids are central to energy metabolism, with the liver playing a key role in regulating lipid homeostasis through anabolic processes such as fatty acid synthesis from glucose, amino acids, and alcohol, and catabolic processes such as mitochondrial fatty acid transport and β‐oxidation (Eliza 2023; Yoon et al. 2021; Enjoji et al. 2016; Nguyen et al. 2008). Disruption of hepatic lipid metabolism is associated with metabolic disorders, including dyslipidemia, metabolic dysfunction‐associated steatotic liver disease (MASLD), and insulin resistance (Farooque et al. 2021; Cetin et al. 2020; Mu et al. 2019).

Functional foods are recognized for their potential to provide health benefits beyond basic nutrition, including the regulation of blood lipid levels (Essa et al. 2023; Sharma and Yadav 2023; Villaño et al. 2016). Most lipid‐lowering functional foods act by inhibiting intestinal cholesterol absorption or hepatic cholesterol synthesis (Jacobo‐Velázquez 2025). However, approaches that enhance lipid utilization and metabolic turnover remain relatively limited (Arner et al. 2019; Smilowitz et al. 2009). Although such effects are commonly observed during exercise and beneficial for maintaining blood lipid levels and body weight (Muscella et al. 2020), functional food components that operate through similar mechanisms are still not well established. Furthermore, despite increasing evidence on individual phytochemicals, the metabolic effects of complex mixtures, particularly from plant‐derived extracts, on hepatic lipid metabolism remain poorly understood.

Citrus is a fruit rich in phytochemicals associated with improved human health outcomes, including effects on lipid management (Nauman and Johnson 2019). Lime ( *Citrus aurantifolia* ) is widely cultivated and consumed worldwide, with major production in countries such as India, China, and Mexico (Villa 2025; ReportLinker Research 2023). Its increasing consumption, particularly in the juice industry, generates substantial peel waste. Citrus peel, which can account for up to half of the fruit weight, represents a potential source of bioactive compounds and an opportunity for value‐added utilization. However, its consumption generates substantial food waste, particularly peel, which accounts for 50% of the weight. Worldwide, the citrus processing industry generates over 60 million tons of waste, necessitating the development of sustainable and innovative solutions for food‐waste utilization (Wedamulla et al. 2022).

To promote sustainable utilization of lime peel, we previously developed a green extraction method to obtain a hesperidin‐ and limonin‐rich extract (Phucharoenrak et al. 2022). Untargeted metabolomics identified more than 60 phytochemicals in lime peel extract, with hesperidin, limonin, and bergaptol as the major constituents (Phucharoenrak et al. 2023). These compounds have been reported to influence lipid metabolism, including lipid uptake, lipogenesis, and fatty acid oxidation (Phucharoenrak and Trachootham 2024; Shylaja et al. 2024; Khorasanian et al. 2023). Despite increasing evidence on individual phytochemicals, the metabolic effects of complex lime peel extracts, particularly on hepatic lipid metabolism, remain poorly understood. A recent in silico study has suggested that compounds from *Citrus aurantifolia* peel may interact with lipid metabolism–related targets, including PCSK9 and HMG‐CoA reductase, supporting their potential role in cholesterol regulation (Sitio et al. 2024). However, experimental evidence at the cellular metabolic level is still limited.

In this study, we hypothesized that lime peel extract enhances lipid catabolism in hepatocytes by modulating mitochondrial fatty acid metabolism. Therefore, we aimed to investigate the effects of lime peel extract on hepatic lipid metabolism in normal human hepatocytes (THLE‐2) using a targeted lipidomics approach. This study is based on in vitro observations and may serve as a basis for further in vivo and clinical investigations.

## 2 Materials and Methods

### 2.1 Materials

Lime peel ethanolic extract was prepared from lime peel powder using the method described in our previous study (Phucharoenrak et al. 2022). Analytical standards of hesperidin (≥ 97.0% purity) and limonin (≥ 95.0% purity) were purchased from Supelco (Bellefonte, PA, USA) and Tokyo Chemical Industry (Tokyo, Japan), respectively. Bronchial Epithelial Cell Growth Medium (BEGM) Bullet Kit was purchased from Lonza Bioscience (Basel, Switzerland). Recombinant human epidermal growth factor (hEGF) was purchased from FUJIFILM Irvine Scientific (Santa Ana, CA, USA). Fetal bovine serum (FBS), 0.25% trypsin, and penicillin/streptomycin were purchased from Gibco (Waltham, MA, USA). 3‐(4,5‐dimethylthiazol‐2‐yl)‐2,5 2,5‐diphenyltetrazolium bromide (MTT), ammonium acetate in methanol, dimethyl sulfoxide (DMSO), phosphate‐buffered saline (PBS), phosphoethanolamine, phenyl isothiocyanate (PITC), and pyridine were purchased from Sigma‐Aldrich (St. Louis, MO, USA). Acetonitrile, isopropanol, methanol, and formic acid were purchased from Fisher Chemical (Fair Lawn, NJ, USA). Ultrapure water (18.2 MΩ) was obtained from ELGA PURELAB Quest UV (High Wycombe, England). AbsoluteIDQ p180 targeted metabolomics kit was purchased from Biocrates (Innsbruck, Austria).

### 2.2 Solvent Selection for Extract Dissolution

From our previous study (Phucharoenrak et al. 2023), the chemical composition of lime peel extract was characterized, with hesperidin and limonin identified as major constituents. Not only are these compounds abundant in lime peel, but they are also reported to play a role in modulating lipid accumulation and fatty acid metabolism (Phucharoenrak and Trachootham 2024; Shylaja et al. 2024; Khorasanian et al. 2023). Therefore, these compounds were selected as markers for solvent selection. Deionized water, 50% DMSO, and pure DMSO were evaluated as solvents for extract dissolution. Solvent suitability was assessed primarily by the recovery of hesperidin and limonin. Quantitation of these compounds was performed after dissolving the lime peel extract in different solvents. Quantitative analysis was performed using UPLC–MS/MS as previously described (Phucharoenrak et al. 2022). Briefly, UHPLC was performed using a Hypersil GOLD C18 column (100 mm × 2.1 mm, 1.9 μm particle size) at 40°C on a Thermo Fisher Scientific system (Waltham, MA, USA). A gradient elution was carried out using 0.1% formic acid in deionized water and 0.1% formic acid in methanol at a flow rate of 0.3 mL/min. Mass spectrometric analysis was conducted using electrospray ionization in positive mode. The precursor ions for hesperidin and limonin were 611.25 and 471.17 m/z, respectively, while the product ions used for quantification and confirmation were 303.14 and 465.208 m/z for hesperidin and 425.21 and 367.1 m/z for limonin, respectively.

### 2.3 Cell Culture

THLE‐2 cells are immortalized human adult hepatocytes. They were obtained from the American Type Culture Collection (ATCC, Manassas, VA, USA). Cells were cultured in BEGM supplemented with high protein bovine pituitary extract, recombinant human insulin, hydrocortisone, retinoic acid, transferrin, hEGF, phosphoethanolamine, penicillin, streptomycin, and 10% heat‐inactivated FBS at 37°C and 5% CO_2_ incubator. The cultured cells were routinely monitored every 2–3 days under an inverted microscope, subcultured, and the culture medium was replaced twice per week.

### 2.4 Cell Viability Assay

THLE‐2 cells were seeded into 96‐well plates and incubated overnight. The cells were then treated with lime peel ethanolic extract at defined concentrations (0, 5, 25, 50, 100, 200, 400, and 500 μg/mL) and incubated further for 24, 48, and 72 h. The concentration range was selected based on our study of lime peel extract in liver cancer cells (Phucharoenrak et al. 2023). It covers both toxic and nontoxic conditions. Each experiment was performed in triplicate, with at least three independent experiments. After incubation, the medium was removed, and the cells were washed with PBS. Subsequently, 50 μL of MTT solution (5 mg/mL in PBS) was added to each well, and the cells were incubated for 3 h at 37°C. Finally, the MTT solution was removed, and the formazan crystals were dissolved in 150 μL of DMSO per well. Cell viability was determined by measuring absorbance at 570 nm with a microplate reader, and the percentage of cell viability was calculated as the ratio of the absorbance of treated cells to that of control cells (0.1% DMSO‐treated).

### 2.5 Targeted Metabolomics Analysis

Cell preparation was adapted according to the company's recommendations and prior studies (Granit et al. 2022; Brunelli et al. 2016). The condition, likely to induce a biological effect but not toxicity, was chosen for the targeted metabolomics study. According to the cell viability result reported in this study, the lime peel at 500 μg/mL caused changes in cell viability in a time‐dependent manner (with viability ranging from over 70% at 24 h to 50% at 72 h). Therefore, this concentration was selected. To ensure a nontoxic condition, a shorter incubation period of 6 h was used based on a comparable number of viable cells compared to the non‐treated control. THLE‐2 cells were seeded in 10 cm culture dishes and treated with an ethanolic extract of lime peel at 500 μg/mL. After incubation for 6 h, the cell pellets were harvested with scrapers and resuspended in isopropanol and phosphate‐buffered saline at a concentration of 1 × 10^6^ cells per 25 μL. This extraction was performed by sonication at 4°C for 3 min, followed by snap‐freezing in liquid nitrogen for 30 s. Subsequently, the cell extracts were centrifuged, and the supernatant was collected for analysis using the AbsoluteIDQ p180 kit (Biocrates Life Sciences AG, Austria).

In brief, 10 μL of cell extracts were seeded into a 96‐well plate and evaporated to dryness under nitrogen. Processed along with the cell extract samples, there were blank, mixed metabolite standard solutions for the calibration curve, and low‐, medium‐, and high‐level quality control (QC) samples. The samples were then derivatized with 50 μL of 5% PITC for 20 min, evaporated to dryness again under nitrogen, and extracted with 300 μL of 5 mM ammonium acetate in methanol for 30 min. Finally, the cell extracts were separated for analysis using liquid chromatography tandem mass spectrometry (LC–MS/MS) with an Ultimate 3000 connected to a TSQ Quantis triple quadrupole mass spectrometer equipped with a Synchronis aQ C18 column (50 mm × 2.1 mm, 1.7 μm particle size, pore size 100 Å) (Thermo Scientific, Waltham, MA, USA) and flow injection analysis tandem mass spectrometry (FIA‐MS/MS) using the TSQ Quantis triple quadrupole mass spectrometer without a column. Raw LC–MS/MS data were quantified using X‐Calibur software (Thermo Scientific, Waltham, MA, USA) and then exported to MetIDQ software (Biocrates Life Sciences AG, Austria). Additionally, raw data from FIA‐MS/MS were exported and quantified using MetIDQ software. QC sample validation (high, medium, and low concentration) was completed before concentration analysis from the calibration curve of each metabolite. Concentration was displayed in μM.

### 2.6 Statistical Analysis

For the cell viability assay, statistical analyses were performed using GraphPad Prism V.9 (GraphPad Software, San Diego, CA, USA). The data were analyzed using parametric statistical tests following assessment of homogeneity of variance and normality across experimental replicates. Analysis of variance (ANOVA) with Dunnett's or Tukey's multiple‐comparison post hoc test was used to compare means among groups. Bartlett's Test was used to determine homogeneity of variance to ensure the correct selection of the statistical test. IC_50_ values were determined from a regression equation obtained through a fitted nonlinear regression model. For the targeted lipidomic analysis, statistical analyses were performed using MetaboAnalyst V.5.0 (Xia Lab, McGill University, Montreal, QC, Canada). *T*‐tests and fold‐change analysis were used to identify key metabolites with significant changes. Principal component analysis (PCA), partial least squares discriminant analysis (PLS‐DA), and orthogonal partial least squares discriminant analysis (OPLS‐DA) were performed to compare the lime peel extract with the control group and to identify key discriminative metabolites. Model performance was validated using fivefold cross‐validation with satisfactory accuracy (1.0), explained variance (*R* ^2^ of 0.97–1.0), and excellent predictive power (*Q* ^2^ of 0.91–1.0). The very small gap between *R* ^2^ and *Q* ^2^ (< 0.1) suggests that the model was not overfitting. Identification of the relevant metabolic pathways associated with dominant metabolites was performed using established lipid metabolism databases, including the Human Metabolome Database (HMDB), LIPID MAPS, and Brejchova et al. (2024).

## 3 Results

### 3.1 Solvent Selection

**FIGURE 1:**
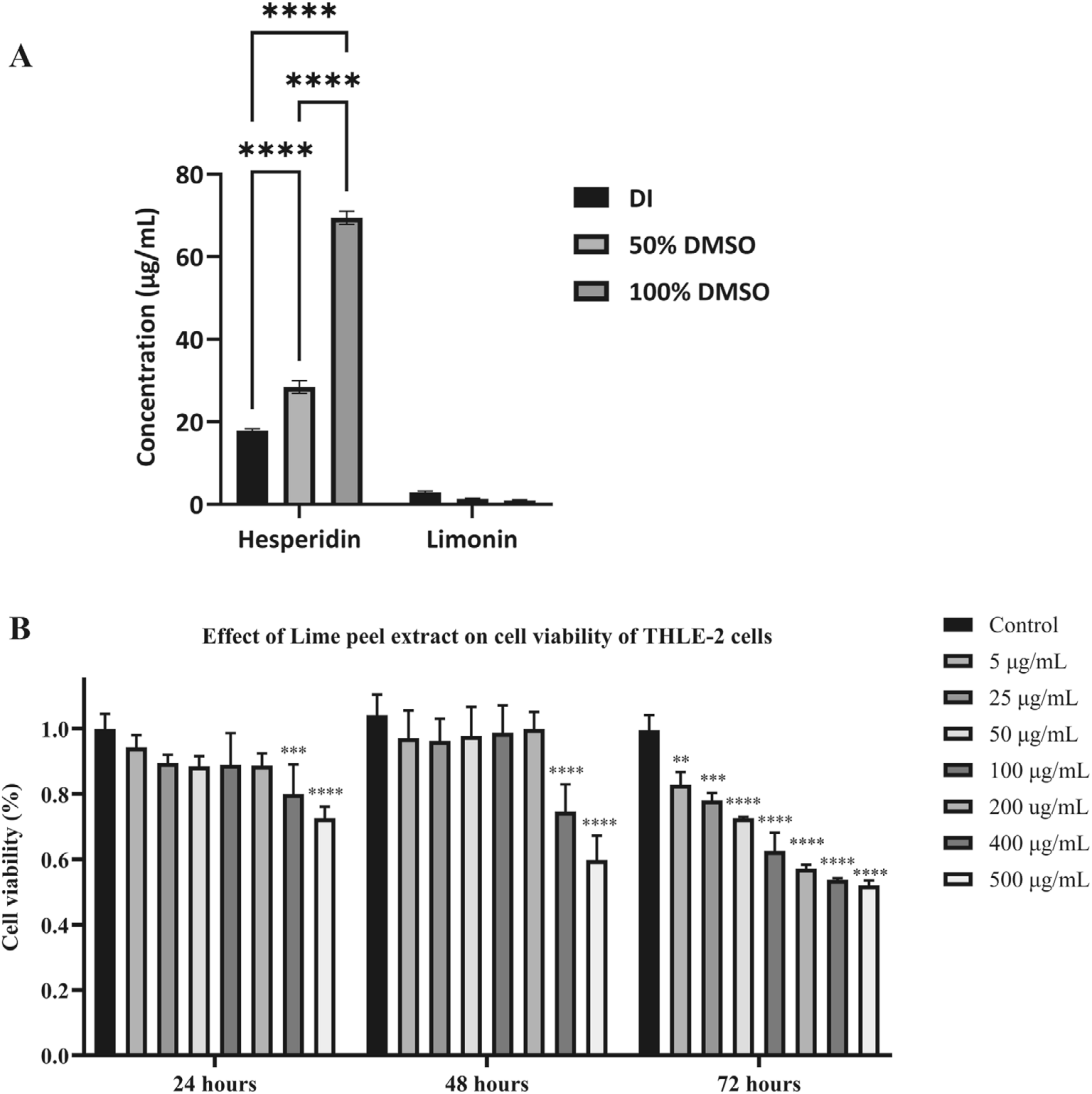
Optimization of the conditions of lime peel extract. (A) Bar graph shows the effect of various solvent conditions (DI, 50% DMSO, and 100% DMSO) on the concentrations of hesperidin and limonin in lime peel extract solution. **** indicates a *p* value of < 0.0001, analyzed by using two‐way ANOVA followed by Tukey's multiple comparisons post hoc test. No symbol indicates no significant difference. (B) Bar graph shows the effect of various doses (5–500 μg/mL) of lime peel ethanolic extract on % cell viability of THLE‐2 human hepatocyte cells treated for 24, 48, and 72 h. **, ***, and **** represent *p* values of < 0.01, < 0.001, and < 0.0001, respectively, compared to the negative control (0.5% DMSO), analyzed by using ANOVA followed by Dunnett's post hoc test.

Lime peel extracts were dissolved in different solvents at 1 g/mL, and the UPLC‐MS/MS technique measured their hesperidin and limonin content. Calibration curves for hesperidin and limonin were linear, with average *R* ^2^ values of 0.9977 and 0.9999, respectively (Figure S1). The highest hesperidin concentration was observed in the extract dissolved in pure DMSO (69.49 ± 1.59 μg/mL), decreasing with increasing water content in the solvent (Figure 1A). In the sample dissolved in deionized water, the hesperidin concentration was significantly lower (17.83 ± 0.56 μg/mL; *p* < 0.0001). In contrast, the highest limonin concentration was found in the aqueous‐dissolved sample (2.90 ± 0.39 μg/mL), although no statistically significant differences were observed between the different solvents (*p* > 0.05) (Figure 1A). Therefore, DMSO was chosen as the solvent for dissolving the lime peel extract in cell model experiments.

### 3.2 Cell Viability

THLE‐2 cells were treated with a range of lime peel ethanolic extract at defined concentrations (0, 5, 25, 50, 100, 200, 400, and 500 μg/mL) for 24, 48, and 72 h. The cytotoxicity of the extract on THLE‐2 cells is shown in Figure 1B. A significant decrease in cell viability was observed at 400 μg/mL after 24 (*p* < 0.001) and 48 h (*p* < 0.0001), and at 500 μg/mL after both 24 and 48 h (*p* < 0.0001). At 72 h, a significant reduction in viability was observed starting at 5 μg/mL (*p* < 0.01 to *p* < 0.0001). Overall, the effect increased with both concentration and incubation time. The IC_50_ for the extract's cytotoxicity on THLE‐2 cells was > 500 μg/mL, with an estimated IC_50_ of 560 μg/mL at 72 h (95% CI). A reduction in cell viability was already detectable at 24 h relative to the control. In contrast, exposure to 500 μg/mL for a shorter incubation time (6 h) yields a comparable number of viable cells to the non‐treated control.

### 3.3 Targeted Lipidomics

**FIGURE 2:**
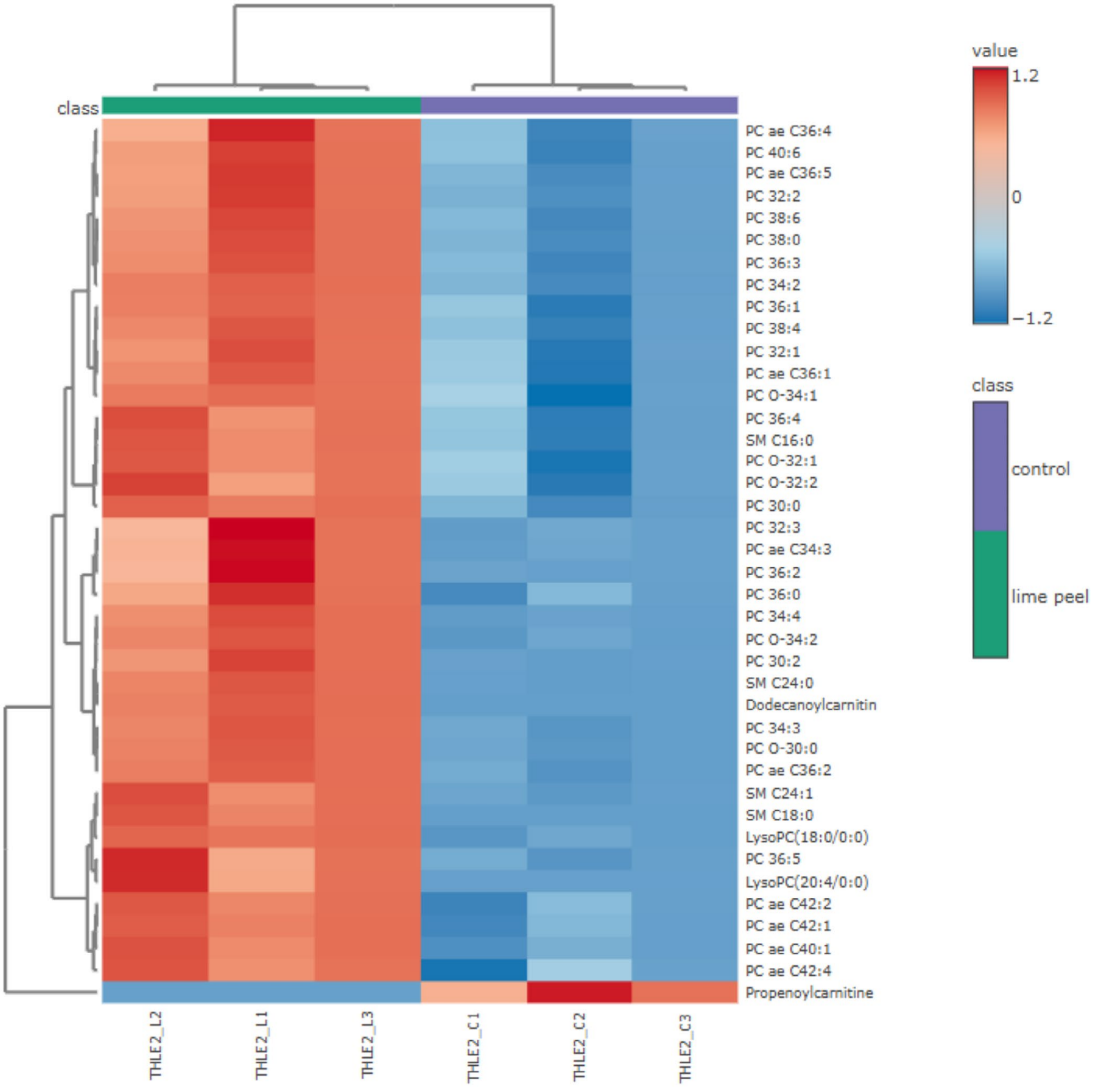
A heat map of significantly altered metabolites in THLE‐2 human hepatocyte cells exposed to lime peel extract (green bar) or control (purple bar), generated by the average linkage and Euclidean distance measurement method. The colors in the heat map represent log‐transformed values of concentration, with red indicating an increase and blue indicating a decrease in metabolite concentration. Triplicate experiments in each group were displayed as labeled at the bottom.

**FIGURE 3:**
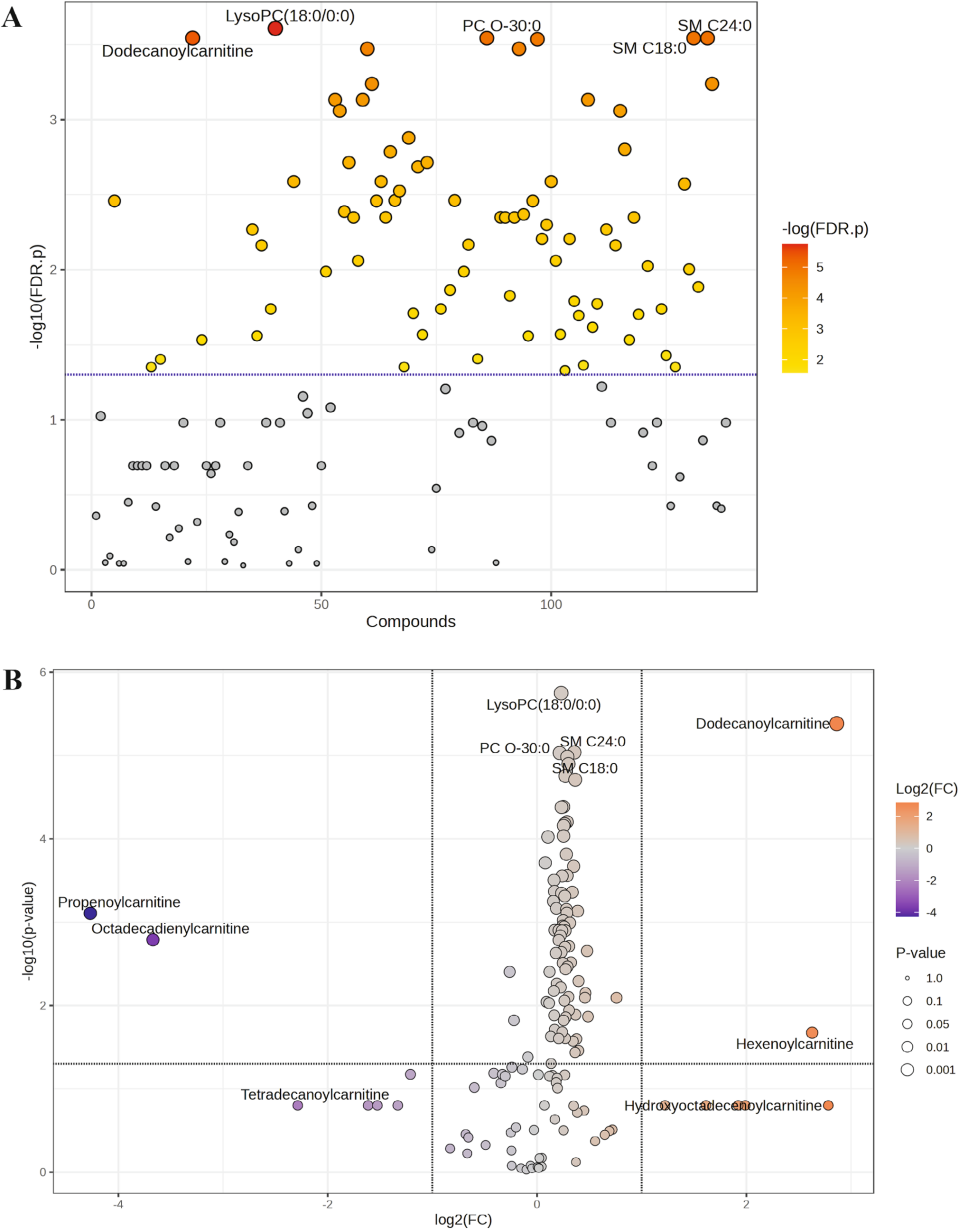
Changes of lipid metabolites in THLE‐2 cells after exposure to lime peel extract in comparison with those of control cells, analyzed by the *t*‐test. (A) Scatter plot of 79 compounds with a statistically significant change (*p* < 0.05). (B) Volcano plot shows four metabolites with at least twofold significant change (*p* < 0.05). Red dot = increased metabolites, blue dot = decreased metabolites.

**FIGURE 4:**
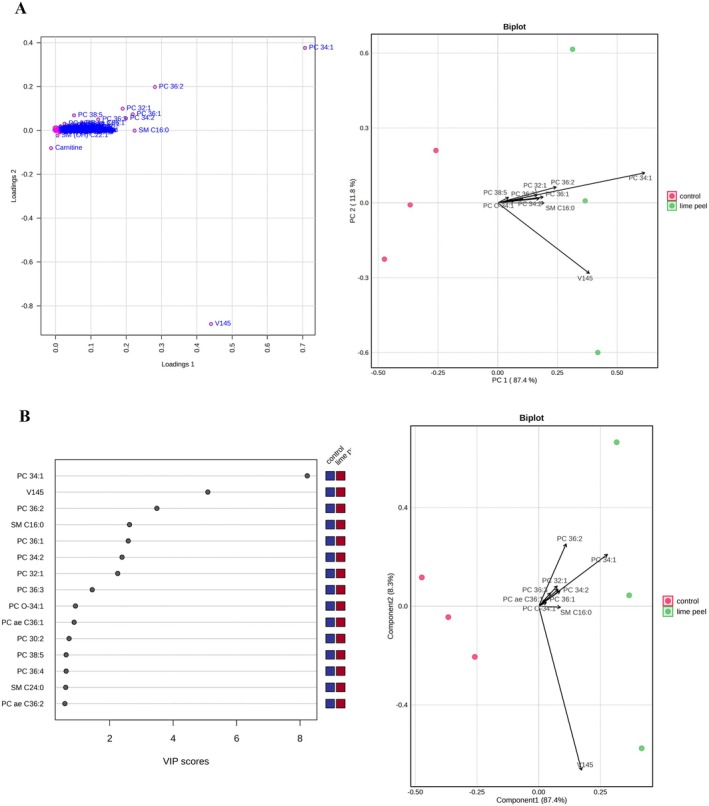
PCA and PLS‐DA analysis. (A) Loading plot and biplot from PCA analysis show metabolites contribute to the difference between lime peel extract and control; the more top right corner indicates more influential. (B) VIP‐plot and biplot from PLS‐DA show ranking of metabolites with higher VIP scores indicating more influential metabolites to predict response (V145 is just default positive control in the software).

**FIGURE 5:**
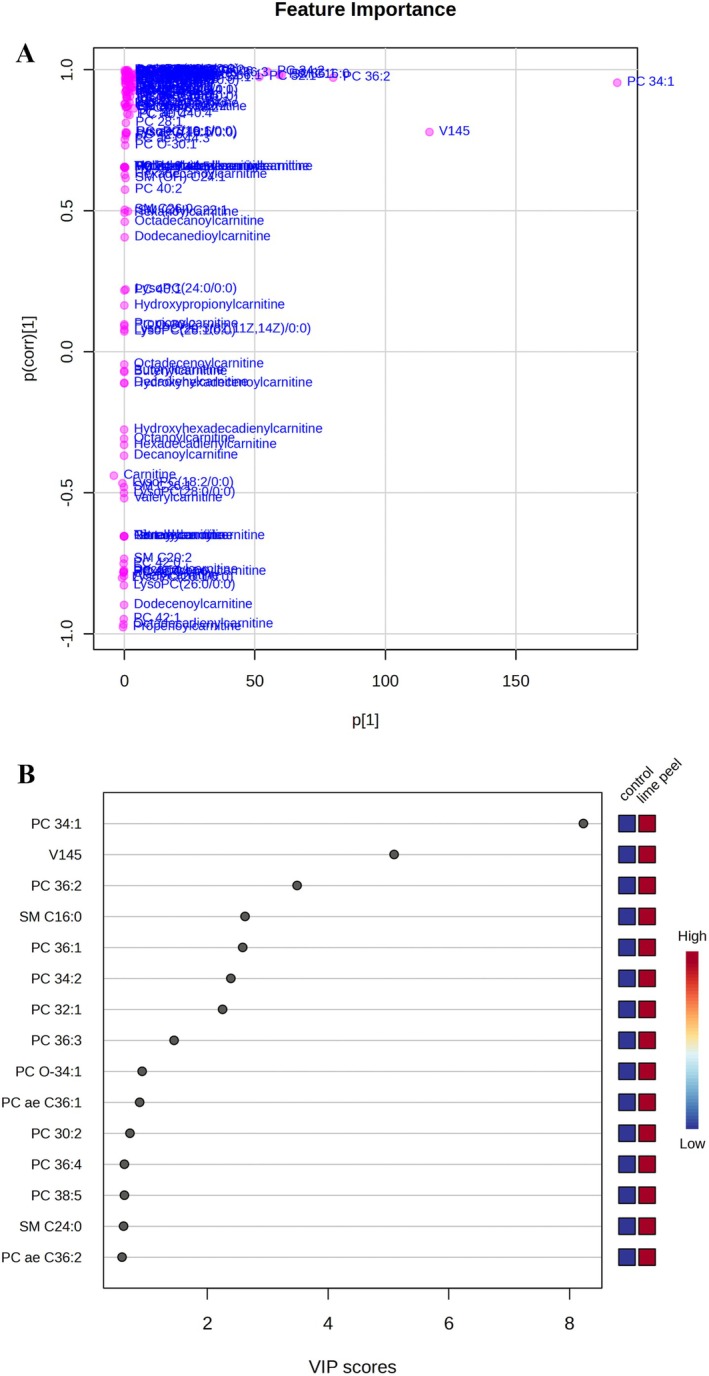
OPLS‐DA analysis. (A) S‐plot shows metabolites contribute to the difference between lime peel extract and control: the more top right corner indicates more influential. (B) VIP‐plot shows ranking of metabolites with higher VIP scores indicating more influential metabolites to predict response.

After exposure to noncytotoxic condition (500 μg/mL of lime peel extract for 6 h), the THLE‐2 cells were prepared for targeted lipidomics using the AbsoluteIDQ p180 kit and analyzed by FIA/MS for 40 acylcarnitine metabolites, 14 glycerophospholipid lysophosphatidylcholines (LysoPC), 36 glycerophospholipid phosphatidylcholines (PC or PC aa), and 38 glycerophospholipid phosphatidylcholines ae (PC ae), and 14 sphingolipid (SM) metabolites. QC sample validation (high, medium, and low concentrations) passed with accuracies ranging from 74.6% to 127.4% (Figure S2). The heat map showed differences in lipid metabolites in THLE‐2 cells between lime peel extract‐treated and control groups, with a trend toward increasing many lipid metabolites (Figure 2). Statistical analyses using the *t*‐test reveal that 79 metabolites are significantly changed (*p* < 0.05 and false discovery rate [FDR] < 0.05) after exposure to lime peel extract, with 75 increased (some acylcarnitine, PC, LysoPC, and SM) and four decreased metabolites (propenoylcarnitine, octadecadienylcarnitine, PC 42:1, and dodecenoylcarnitine) (Figure 3A, Tables S1 and S2). Among those metabolites, LysoPC (18:0/0:0), SM C24:0, PC O‐30:0, and dodecanoylcarnitine showed a highly significant increase (*p* < 1 × 10^−6^), while propenoylcarnitine showed a significant decrease (*p* < 0.001) (Table S1, Figure S3). Fold‐change analysis revealed that all metabolites exhibiting at least a twofold change were acylcarnitines (Table 1). A significant over twofold increase (*p* < 0.05) was observed in medium‐chain acylcarnitines (dodecanoylcarnitine [C12] and hexenoylcarnitine [C6:1]), while a decrease was found in long‐chain acylcarnitine (octadecadienylcarnitine: C18:2) (Figure 3B, Table 2). Furthermore, a significant reduction (*p* < 0.05) in propenoylcarnitine (C3:1) was observed (Figure 3B, Table 2). While dodecanoylcarnitine (C12) increased about fourfold upon exposure to lime peel extract, the octadecadienylcarnitine (C18:2) decreased almost fourfold (Figure S4). PCA, PLS‐DA, and OPLS‐DA consistently reveal phosphatidylcholine (PC) 34:1 at the top right‐most position of the PCA loading and biplots, and S‐plot in both PLS‐DA and OPLS‐DA analyses, with the VIP score at 8 and 12, respectively (Figures 4 and 5). PC 34:1 was significantly increased upon exposure to lime peel extract (*p* = 0.003; Table S1).

**TABLE 1:** Metabolites with at least a twofold difference between the lime peel extract‐treated cells and those of the control, analyzed by fold‐change analysis.

Metabolites	Fold change	log_2_ (FC)
**Increased**		
Dodecanoylcarnitine (C12)	7.2727	2.8625
Hydroxyoctadecenoylcarnitine (C18:1‐OH)	6.8788	2.7822
Hexenoylcarnitine (C6:1)	6.1818	2.628
Tiglylcarnitine (C5:1)	3.9697	1.989
Methylglutarylcarnitine (C5‐M‐DC)	3.7879	1.9214
Hydroxyvalerylcarnitine (C5‐OH)	3.0606	1.6138
Hydroxyhexadecanoylcarnitine (C16‐OH)	2.3333	1.2224
**Decreased**		
Propenoylcarnitine (C3:1)	0.051887	−4.2685
Octadecadienylcarnitine (C18:2)	0.078571	−3.6699
Tetradecanoylcarnitine (C14)	0.20497	−2.2865
Nonaylcarnitine (C9)	0.32673	−1.6138
Glutarylcarnitine (C5‐DC)	0.34737	−1.5255
Pimeloylcarnitine (C7‐DC)	0.39759	−1.3306
Hexadecenoylcarnitine (C16:1)	0.43237	−1.2097

**TABLE 2:** Lipid metabolites in THLE‐2 cells with at least twofold changes and statistically significant (*p* < 0.05) after exposure to lime peel extract.

Metabolites	Fold changes (FC)	log_2_ (FC)	*p*	log_10_ (*p*)
**Increased**				
Dodecanoylcarnitine (C12)	7.2727	2.8625	0.000286	3.5438
Hexenoylcarnitine (C6:1)	6.1818	2.628	0.039521	1.4032
**Decreased**				
Propenoylcarnitine (C3:1)	0.051887	−4.2685	0.003481	2.4583
Octadecadienylcarnitine (C18:2)	0.078571	−3.6699	0.005392	2.2683

## 4 Discussion

Management of dyslipidemia involves a combination of diet and exercise. Most functional foods with lipid‐lowering properties (Hunter and Hegele 2017) help maintain the lipid profile by inhibiting dietary cholesterol absorption, as with plant sterols or soluble fiber (Hughes and Grafenauer 2021; Ras et al. 2014; Sood et al. 2008), or by inhibiting cholesterol synthesis, as with monacolin K, brutieridin, and melitidin (Rahmani et al. 2023; Nauman and Johnson 2019). While exercise has been known to increase fat metabolism, functional foods that improve lipid metabolism are rare. Our current metabolomics study reveals that a hesperidin‐limonin‐bergaptol‐rich lime ( *Citrus aurantifolia* ) peel extract increases medium‐chain acylcarnitines and decreases long‐chain acylcarnitines, which may indicate potential changes in fatty acid transport and mitochondrial β‐oxidation. This dominant medium‐chain acylcarnitine metabolomic pattern is similar to that observed during exercise (Lehmann et al. 2010). Furthermore, exposure to the extract results in a significant decrease in propropenoylcarnitine (C3:1). Since this metabolite is an important intermediate in the synthesis of fatty acids from branch‐chain amino acids (Brejchova et al. 2024), one could speculate that when the extract is taken with protein‐containing foods, it may shift amino acid metabolism toward muscle protein synthesis rather than fat deposition. Further studies are required to confirm this hypothesis and the underlying mechanisms require further investigation. Since the catabolism of lipids is a crucial process in regulating body weight by breaking down stored fats for energy (Arner et al. 2019; Smilowitz et al. 2009), the modulatory effect of lime peel extract on lipid transport and metabolism may have potential implications for maintaining blood lipid levels and body weight. Future studies are warranted to elucidate its clinical efficacy in lipid lowering. In this study, PCA, PLS‐DA, and OPLS‐DA analyses consistently identified phosphatidylcholine 34:1 (PC34:1) as the discriminative metabolite associated with treatment response, with the highest contribution, as indicated by the VIP score. Low levels of PC 34:1 in liver cells are strongly linked to the progression of nonalcoholic steatohepatitis (NASH) from simpler fatty liver (NAFLD), and supplementation of PC can reduce liver injury, inflammation, and oxidative stress (Tian et al. 2024; Wattacheril et al. 2013). The observed increase of PC 34:1 in liver cells after treatment with lime peel extract may indicate a potential protective role against metabolic dysfunction‐associated steatotic liver disease (MASLD) (Zhu et al. 2024), warranting further study.

In general, acylcarnitine plays a crucial role in cellular energy metabolism, particularly in the liver (Schooneman et al. 2015). Acylcarnitine pathways involve β‐oxidation of various fatty acids, the ketogenic pathway, and amino acid metabolism, including branched‐chain amino acids and other amino acids (Brejchova et al. 2024). In this study, metabolites showing at least twofold changes after exposure to lime peel extract are all acylcarnitines involved in β‐oxidation and branched‐chain amino acid metabolism. As key intermediates in mitochondrial fatty acid transport and oxidation, alterations in acylcarnitine levels may reflect changes in lipid catabolism (Brejchova et al. 2024). Dodecanoylcarnitine (C12) and hexenoylcarnitine (C6:1) are medium‐chain acylcarnitines, participating in mitochondrial transport and the β‐oxidation pathway of straight fatty acids. Octadecadienylcarnitine (C18:2) is a long‐chain acylcarnitine, participating in mitochondrial transport and the β‐oxidation pathway of straight polyunsaturated fatty acids (Brejchova et al. 2024). Therefore, the significant increases in medium‐chain (C12 and C6:1) and decreases in long‐chain (C18:2) acylcarnitines may reflect alterations in fatty acid oxidation following treatment with lime peel extract. Interestingly, a clinical study reported that an increase in medium‐chain acylcarnitines was the predominant metabolomic pattern during moderately intense exercise, supporting lipid oxidation (Lehmann et al. 2010). To our knowledge, the exercise‐like lipidomic pattern has not been reported in other functional food ingredients before. Future studies to compare the in vivo and clinical effects of lime peel extract with those of exercise are worthwhile.

A previous clinical trial reported increases in short‐chain acylcarnitines and decreases in medium‐ and long‐chain acylcarnitines after 2 weeks of daily orange juice consumption in healthy volunteers (Moreira et al. 2018). This discrepancy with our study regarding the effect on medium‐chain acylcarnitine may be attributable to differences in the predominant phytochemicals in orange juice and lime peel extract. Commonly consumed orange juice (sweet orange) typically contains high levels of hesperidin (up to 90% of total flavones) but very low levels of bitter and sour compounds such as limonin and bergaptol (Zhang et al. 2024; Han et al. 2022; Huang et al. 2018; Silva et al. 2014; Fellers and Hill 1986). In contrast, our lime peel extract is rich in hesperidin, limonin, and bergaptol (Phucharoenrak et al. 2023), which have been reported to be associated with lipid metabolism, including effects on lipid accumulation and fatty acid metabolism (Phucharoenrak and Trachootham 2024; Shylaja et al. 2024; Khorasanian et al. 2023). Previous studies have reported that limonin can activate AMP‐activated protein kinase (AMPK) and upregulate the expression of genes involved in mitochondrial and peroxisomal beta‐oxidation, such as acyl‐CoA oxidase 1 (Acox1) and carnitine palmitoyltransferase 1 (CPT1) (Wang et al. 2022; Fukuchi et al. 2008). Likewise, Mirarchi et al. (2022) demonstrated that bergamot extract, which contains bergaptol, has been shown to enhance fatty acid metabolism by increasing the expression of genes involved in mitochondrial β‐oxidation (e.g., Acox1, Pparα, Ucp2) in liver cells. Mixtures of phytochemicals in different proportions may explain the distinct effects between lime peel extract and orange juice. Synergistic effects of these bioactive compounds likely play a key role in modulating acylcarnitine pathways. Our previous study found a synergistic anticancer effect between hesperidin and limonin compounds (Phucharoenrak et al. 2023). Interestingly, the lime peel extract has a superior effect compared with the combination of hesperidin and limonin, suggesting that a complex mixture of phytochemicals could potentiate efficacy (Phucharoenrak et al. 2023). Although the potential effects of limonin, hesperidin, and bergaptol on lipid metabolism have been reported separately, their synergistic effects have not been well studied and warrant further investigation.

Besides the β‐oxidation pathway, acylcarnitine also plays a key role in amino acid metabolism. When we consume protein or amino acids, they can be used for protein synthesis or converted into keto acids, which are then either entered into the TCA cycle for energy production or further converted into glucose or lipids (Gyamfi and Owusu Danquah 2016). Interestingly, in this study, we observed a significant decrease in propenoylcarnitine (C3:1), a metabolite formed during the breakdown of branched‐chain amino acids, particularly valine and isoleucine, to keto acids, acetyl‐CoA, and acrylyl‐CoA (Brejchova et al. 2024). Therefore, the findings may reflect potential alterations in branched‐chain amino acid–related lipid metabolism following exposure to lime peel extract. Further studies to elucidate amino acid metabolites are warranted to confirm this hypothesis. Furthermore, propenoylcarnitine (C3:1) is classified as an unsaturated short‐chain acylcarnitine, which is associated with oxidative stress, fatty acid oxidation, and energy metabolism (Wishart et al. 2022; Liu et al. 2021). Previous studies have indicated that increased propenoylcarnitine levels are associated with metabolic dysfunction and liver damage (Liu et al. 2021). Thus, a significant decrease in propenoylcarnitine suggests a beneficial effect of lime peel extract.

This study implies the use of MS‐based targeted lipidomics, which enables simultaneous, comprehensive profiling of multiple lipid metabolite classes in hepatocytes, thereby facilitating the identification of metabolic pathways influenced by lime peel extract. This approach reduces false positives, minimizes analytical artifacts, establishes a lower limit of detection, and enables quantification that supports accurate comparisons between study groups. It also allows us to gain molecular insights and propose a potentially related pathway, offering greater precision than untargeted metabolomics. Furthermore, our study investigated metabolomic changes in a normal liver cell line (THLE‐2), whereas most studies use liver cancer cells (HepG2). This allows us to understand the biological effect of lime peel extract on the normal liver, which could be translated into future clinical studies in healthy people. Nevertheless, there are some limitations. Although this study used a highly reliable MS‐based targeted method, the analysis was performed on medium‐resolution MS, which may have lower accuracy in determining mass‐to‐charge ratios (1–2 decimal places) than high‐resolution MS (3–4 decimal places). Furthermore, the findings are based on a cell model study that does not account for changes during the kinetics (absorption, distribution, and metabolism) when the extract is administered to humans. The effect of lime peel extract on lipid metabolism warrants further study in in vivo models and clinical trials.

## 5 Conclusions

The findings of this in vitro study suggest that lime peel extract may reduce lipid synthesis from amino acids and may promote lipid catabolism in hepatocyte cell models, with a dominant medium‐chain acylcarnitine pattern. PC34:1 was identified as a discriminative metabolite for the cellular response to the extract. The preliminary insight suggests that this novel functional ingredient may have potential implications in preventing dyslipidemia and metabolic dysfunction‐associated steatotic liver disease (MASLD), warranting further in vivo and clinical studies.

## Author Contributions

**Pakkapong Phucharoenrak:** conceptualization, methodology, investigation, data curation, formal analysis, visualization, writing – original draft. **Kemika Praengam:** methodology, investigation, data curation, formal analysis. **Dunyaporn Trachootham:** conceptualization, methodology, supervision, formal analysis, funding acquisition, project administration, writing – review and editing.

## Funding

This research is supported by Mahidol University (MU's Strategic Research Fund): 2023.

## Ethics Statement

This manuscript describes an in vitro study. Therefore, it does not require any approval from the ethical committee. Authorship adheres to ethical principles for research conduct.

## Conflicts of Interest

The authors declare no conflicts of interest.

## Supporting information


**Figure S1:** Average calibration curves of hesperidin (A) and Limonin (B) generated by the linear plots between areas under the curves of the quantitative product ions and the concentrations of standard solutions. *R* ^2^ values were obtained from linear regression.
**Figure S2:** QC validation for the p180 metabolomic kit for analysis of targeted lipidomics.
**Figure S3:** Box plots showing the normalized levels (relative abundance) of propenoylcarnitine in THLE‐2 human hepatocyte cells between the control group and the lime peel extract‐treated group. The *y*‐axis represents normalized metabolite abundance.
**Figure S4:** Violin plots comparing the normalized levels (relative abundance) of dodecanoylcarnitine (A) and octadecadienylcarnitine (B) in THLE‐2 human hepatocyte cells between the control group (red) and the treatment group (green). The *y*‐axis represents normalized metabolite abundance.
**Table S1:** Metabolites with a statistically significant increase in THLE‐2 cells after exposure to lime peel extract, analyzed by *t*‐test.
**Table S2:** Metabolite with a statistically significant decrease in THLE‐2 cells after exposure to lime peel extract, analyzed by *t*‐test.

## Data Availability

The data that support the findings of this study are available from the corresponding author upon reasonable request.
