# Supplementary material for: Targeted Lipidomics Reveals Citrus aurantifolia Peel Extract's Potential to Increase Lipid Catabolism With PC34:1 as a Discriminative Metabolite
Source: Food Sci Nutr. 2026 Jul 20;14(7):e72108. doi: 10.1002/fsn3.72108 (PMC13382369; doi:10.1002/fsn3.72108)
Supplement: Supplementary file 1 — Figure S1: Average calibration curves of hesperidin (A) and Limonin (B) generated by the linear plots between areas under the curves of the quantitative product ions and the concentrations of standard solutions. R 2 values were obtained from linear regression. Figure S2: QC validation for the p180 metabolomic kit for analysis of targeted lipidomics. Figure S3: Box plots showing the normalized levels (relative abundance) of propenoylcarnitine in THLE‐2 human hepatocyte cells between the control group and the lime peel extract‐treated group. The y‐axis represents normalized metabolite abundance. Figure S4: Violin plots comparing the normalized levels (relative abundance) of dodecanoylcarnitine (A) and octadecadienylcarnitine (B) in THLE‐2 human hepatocyte cells between the control group (red) and the treatment group (green). The y‐axis represents normalized metabolite abundance. Table S1: Metabolites with a statistically significant increase in THLE‐2 cells after exposure to lime peel extract, analyzed by t‐test. Table S2: Metabolite with a statistically significant decrease in THLE‐2 cells after exposure to lime peel extract, analyzed by t‐test. [file FSN3-14-e72108-s001.docx]

**
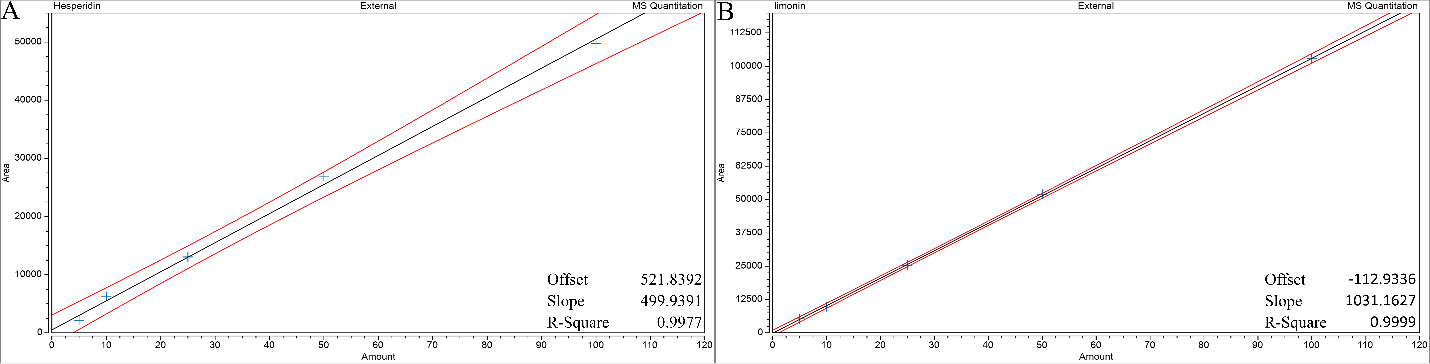
**

**Figure S1** Average calibration curves of hesperidin (**A**) and Limonin (**B**) generated by the linear plots between areas under the curves of the quantitative product ions and the concentrations of standard solutions. R^2^ values were obtained from linear regression.

**Figure S2:** QC validation for the p180 metabolomic kit for analysis of targeted lipidomics


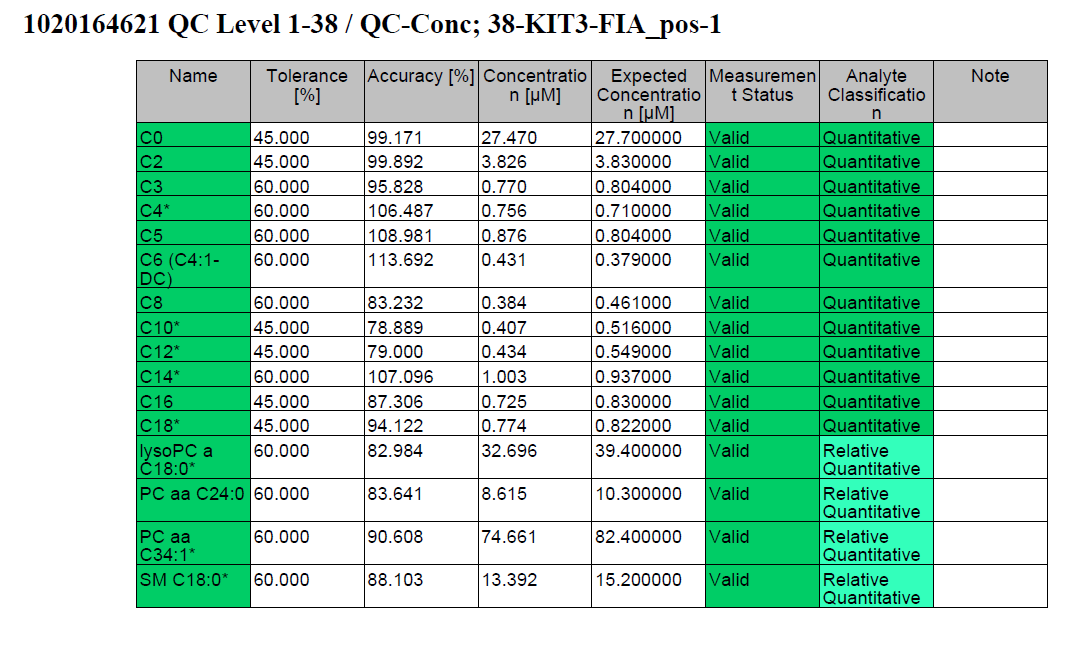


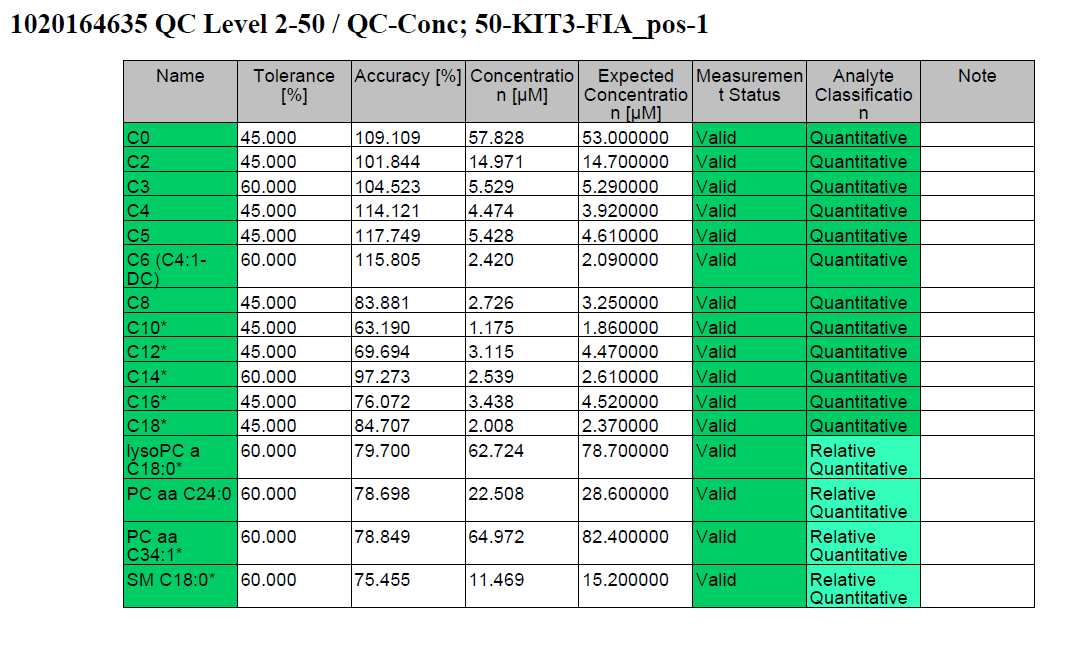


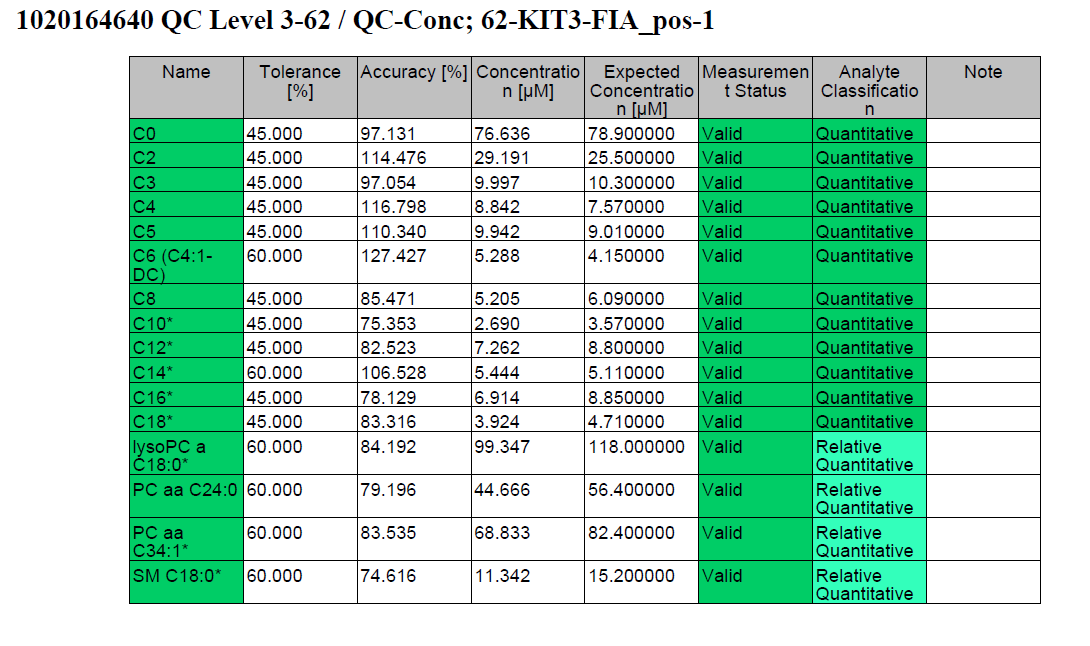


**Figure S3:** Box plots showing the normalized levels (relative abundance) of propenoylcarnitine in THLE-2 human hepatocyte cells between the control group and the lime peel extract-treated group. The y-axis represents normalized metabolite abundance.

**
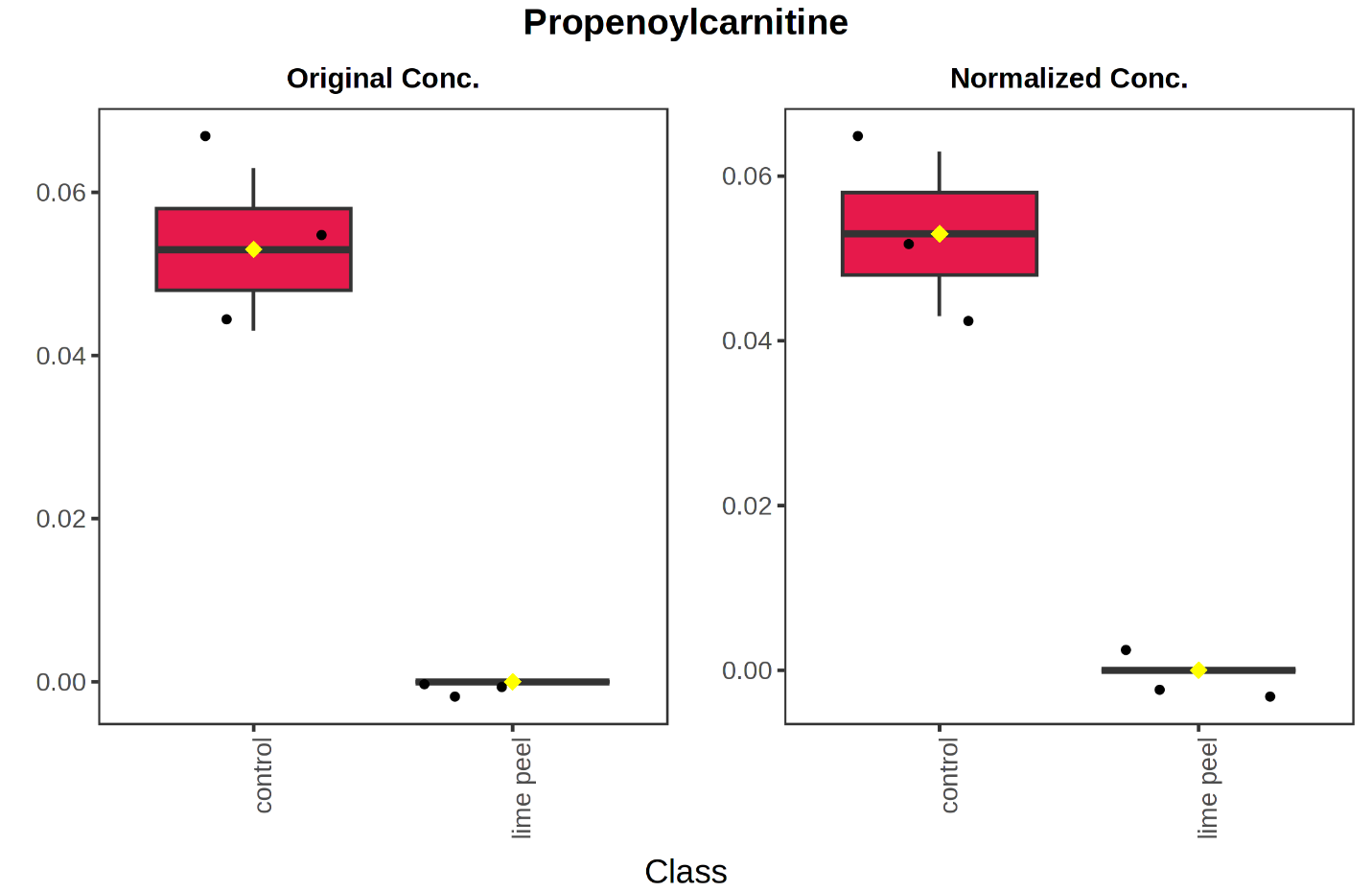
**

**Figure S4:** Violin plots comparing the normalized levels (relative abundance) of dodecanoylcarnitine (A) and octadecadienylcarnitine (B) in THLE-2 human hepatocyte cells between the control group (red) and the treatment group (green). The y-axis represents normalized metabolite abundance.

**
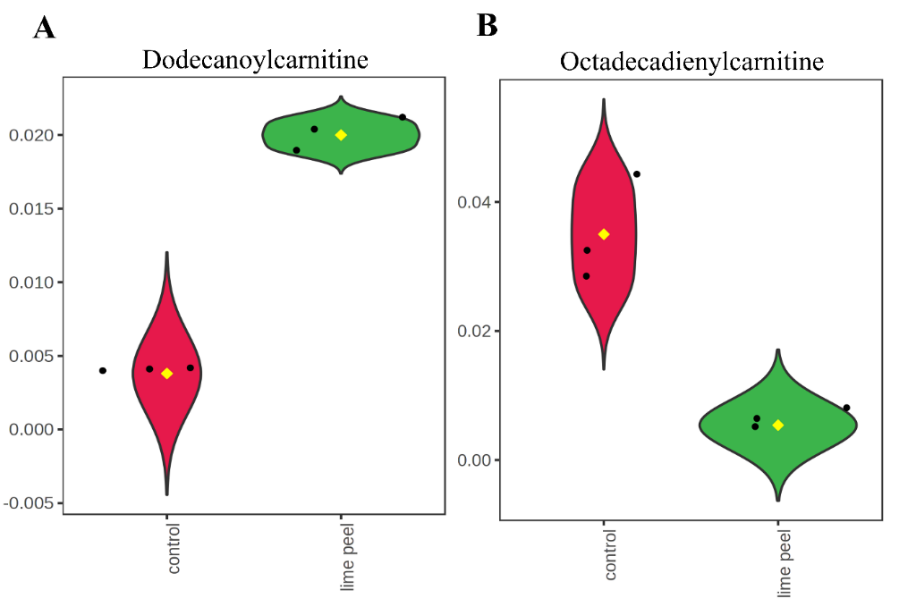
**

**Table S1:** Metabolites with a statistically significant increase in THLE-2 cells after exposure to lime peel extract, analyzed by t-test

| **Metabolite** | **t.stat** | **p.value** | **False discovery rate** |
| --- | --- | --- | --- |
| LysoPC(18:0/0:0) | -42.774 | 1.79E-06 | 0.000246 |
| SM C24:0 | -28.376 | 9.18E-06 | 0.000286 |
| PC O-30:0 | -28.263 | 9.33E-06 | 0.000286 |
| **Dodecanoylcarnitine** | **-28.059** | **9.60E-06** | **0.000286** |
| SM C18:0 | -27.52 | 1.04E-05 | 0.000286 |
| PC ae C36:2 | -26.178 | 1.27E-05 | 0.000291 |
| PC O-34:2 | -24.082 | 1.76E-05 | 0.000337 |
| PC 34:3 | -23.466 | 1.95E-05 | 0.000337 |
| PC 34:4 | -19.441 | 4.13E-05 | 0.000577 |
| SM C24:1 | -19.377 | 4.18E-05 | 0.000577 |
| PC 34:2 | -17.488 | 6.28E-05 | 0.000737 |
| PC ae C40:1 | -17.253 | 6.62E-05 | 0.000737 |
| PC 30:0 | -17.047 | 6.95E-05 | 0.000737 |
| PC 30:2 | -15.847 | 9.27E-05 | 0.000873 |
| PC ae C42:1 | -15.75 | 9.49E-05 | 0.000873 |
| PC 38:0 | -13.947 | 0.000153 | 0.001322 |
| PC ae C42:2 | -13.132 | 0.000194 | 0.001576 |
| PC 36:3 | -12.817 | 0.000214 | 0.001637 |
| PC 38:6 | -11.997 | 0.000277 | 0.001929 |
| PC 32:2 | -11.966 | 0.00028 | 0.001929 |
| PC 38:4 | -11.619 | 0.000314 | 0.002061 |
| PC ae C36:5 | -10.729 | 0.000428 | 0.002586 |
| LysoPC(20:4/0:0) | -10.666 | 0.000438 | 0.002586 |
| PC 36:1 | -10.591 | 0.00045 | 0.002586 |
| SM C16:0 | -10.38 | 0.000486 | 0.002685 |
| PC 36:5 | -9.9924 | 0.000564 | 0.002992 |
| PC 36:4 | -9.5054 | 0.000684 | 0.003458 |
| PC 40:6 | -9.442 | 0.000702 | 0.003458 |
| PC 36:0 | -9.3237 | 0.000737 | 0.003505 |
| PC ae C36:1 | -9.203 | 0.000774 | 0.003563 |
| PC 32:1 | -8.7265 | 0.00095 | 0.004229 |
| PC ae C34:3 | -8.5598 | 0.001023 | 0.004411 |
| PC O-32:1 | -8.3829 | 0.001108 | 0.004601 |
| PC 36:2 | -8.3295 | 0.001135 | 0.004601 |
| PC ae C42:4 | -8.1273 | 0.001247 | 0.004601 |
| PC 32:3 | -8.117 | 0.001253 | 0.004601 |
| PC O-34:1 | -8.1002 | 0.001263 | 0.004601 |
| PC O-32:2 | -8.0931 | 0.001267 | 0.004601 |
| PC ae C36:4 | -7.8042 | 0.001455 | 0.005147 |
| PC ae C40:5 | -7.5597 | 0.001641 | 0.005523 |
| PC ae C36:3 | -7.2256 | 0.001946 | 0.006378 |
| PC ae C38:3 | -7.1854 | 0.001987 | 0.006378 |
| PC 42:2 | -6.9772 | 0.002219 | 0.00696 |
| PC ae C42:0 | -6.9156 | 0.002294 | 0.007035 |
| LysoPC(16:0/0:0) | -6.8748 | 0.002345 | 0.007036 |
| PC ae C38:0 | -6.407 | 0.003049 | 0.00872 |
| **PC 34:1** | **-6.3803** | **0.003096** | **0.00872** |
| PC ae C44:4 | -6.2092 | 0.003423 | 0.009448 |
| SM C16:1 | -6.0933 | 0.003669 | 0.009927 |
| PC 26:0 | -5.9752 | 0.003942 | 0.010285 |
| SM C18:1 | -5.5681 | 0.005097 | 0.013024 |
| PC 40:5 | -5.4651 | 0.005452 | 0.013679 |
| PC O-34:0 | -5.307 | 0.006059 | 0.01493 |
| PC ae C38:4 | -5.1616 | 0.006691 | 0.016198 |
| PC ae C40:3 | -5.0776 | 0.007092 | 0.016875 |
| SM (OH) C14:1 | -4.9297 | 0.007875 | 0.018265 |
| PC40:3 | -4.8979 | 0.008056 | 0.018265 |
| LysoPC(17:0/0:0) | -4.8949 | 0.008074 | 0.018265 |
| PC 38:3 | -4.7825 | 0.008759 | 0.019497 |
| PC ae C42:5 | -4.7395 | 0.00904 | 0.019802 |
| PC ae C38:5 | -4.6924 | 0.009361 | 0.020185 |
| PC ae C40:2 | -4.4347 | 0.011381 | 0.024162 |
| PC ae C38:1 | -4.2753 | 0.012896 | 0.026964 |
| PC 38:5 | -4.251 | 0.013148 | 0.027081 |
| LysoPC(14:0/0:0) | -4.2093 | 0.013593 | 0.027586 |
| PC ae C36:0 | -4.1888 | 0.013819 | 0.027639 |
| PC ae C42:3 | -4.0783 | 0.015119 | 0.029386 |
| SM (OH) C16:1 | -3.7808 | 0.019423 | 0.037228 |
| PC 42:5 | -3.7044 | 0.020755 | 0.039235 |
| **Hexenoylcarnitine** | **-3.6806** | **0.021193** | **0.039521** |
| PC ae C38:6 | -3.5629 | 0.023525 | 0.043871 |
| PC 36:6 | -3.5085 | 0.024705 | 0.045053 |
| SM (OH) C22:2 | -3.5037 | 0.024812 | 0.045053 |
| Glutaconylcarnitine | -3.4887 | 0.025153 | 0.045079 |
| PC ae C38:2 | -3.418 | 0.026829 | 0.047466 |

**Table S2: Metabolite with a statistically significant decrease in THLE-2 cells after exposure to lime peel extract, analyzed by t-test**

| **Metabolite** | **t.stat** | **p.value** | **False discovery rate** |
| --- | --- | --- | --- |
| Propenoylcarnitine | 7.6903 | 0.001538 | 0.005306 |
| Octadecadienylcarnitine | 6.4086 | 0.003046 | 0.00872 |
| PC 42:1 | 5.9718 | 0.00395 | 0.010285 |
| Dodecenoylcarnitine | 4.0819 | 0.015075 | 0.029386 |
